# Unveiling the Effects of Triptorelin on Endocrine Profiles: Insights From Healthy, Polycystic Ovary Syndrome, and Hypothalamic Amenorrhea Women

**DOI:** 10.7759/cureus.44752

**Published:** 2023-09-05

**Authors:** Riya Kumari, Komal N Muneshwar, Aniket G Pathade, Seema Yelne

**Affiliations:** 1 Community Medicine, Jawaharlal Nehru Medical College, Datta Meghe Institute of Higher Education and Research, Wardha, IND; 2 Research and Development, Jawaharlal Nehru Medical College, Datta Meghe Institute of Higher Education and Research, Wardha, IND; 3 Nursing, Shalinitai Meghe College of Nursing, Datta Meghe Institute of Higher Education and Research, Wardha, IND

**Keywords:** personalized treatment, women’s health, hormonal regulation, reproductive health, hypothalamic amenorrhea, polycystic ovary syndrome, endocrine profiles, gonadotropin-releasing hormone agonist, triptorelin

## Abstract

Triptorelin, a synthetic gonadotropin-releasing hormone (GnRH) agonist, has garnered increasing attention for its profound effects on endocrine profiles across diverse populations. This review article explores triptorelin’s impact on women’s health by examining its effects on healthy individuals, those with polycystic ovary syndrome (PCOS), and those experiencing hypothalamic amenorrhea (HA). The mechanism of triptorelin involves a transient surge in gonadotropin release, followed by receptor desensitization, leading to downregulation of the hypothalamus-pituitary-gonadal (HPG) axis. In healthy women, triptorelin’s controlled modulation of the HPG axis is a foundation for assisted reproduction techniques. In PCOS, it offers promise in restoring ovulatory function and mitigating hyperandrogenism. For HA individuals, triptorelin’s potential to restore proper GnRH pulsatility emerges as a therapeutic avenue. This review emphasizes the importance of personalized approaches based on specific health conditions, highlighting triptorelin’s versatility and potential applications beyond its current scope. As research progresses, triptorelin’s role in endocrine management is poised to reshape women’s health by optimizing hormonal equilibrium and overall well-being.

## Introduction and background

The intricate dance of hormones within the human body orchestrates a symphony of physiological processes, including reproduction, metabolism, growth, and overall well-being. The hypothalamus-pituitary-gonadal (HPG) axis is central to this hormonal symphony, a complex network responsible for regulating the endocrine system’s key players. One notable component of this axis is triptorelin, a synthetic peptide analog of gonadotropin-releasing hormone (GnRH). In recent years, triptorelin has garnered significant attention for its ability to modulate endocrine profiles, affecting various health conditions. This review delves into the multifaceted effects of triptorelin on endocrine profiles, shedding light on its implications for healthy women, those with polycystic ovary syndrome (PCOS), and those experiencing hypothalamic amenorrhea (HA) [1,2].

Triptorelin, classified as a GnRH agonist, exerts its effects by binding to and activating GnRH receptors in the pituitary gland. This leads to a transient surge in gonadotropin release, desensitizing these receptors and causing downregulation of gonadotropin secretion. By manipulating the pulsatile release of GnRH, triptorelin can effectively suppress the gonadotropins luteinizing hormone (LH) and follicle-stimulating hormone (FSH). This suppression holds therapeutic potential in various contexts, including infertility treatment, sex hormone-related cancers, and hormone-dependent conditions [3,4].

Endocrine profiles serve as windows into the intricate balance of hormones within the body. Deviations from these profiles can herald a wide array of health concerns. Hormones are pivotal in maintaining menstrual regularity, promoting ovulation, regulating metabolic processes, and modulating mood and cognition. Therefore, disturbances in endocrine profiles can profoundly affect reproductive health, metabolic homeostasis, and overall quality of life. Understanding these profiles facilitates diagnosis and monitoring and enables the development of targeted interventions to restore equilibrium [5-7].

Studying the effects of triptorelin on endocrine profiles across different populations provides valuable insights into its potential applications and mechanisms of action. Healthy women with regular menstrual cycles are a baseline for assessing triptorelin’s impact on normal endocrine regulation. Investigating triptorelin’s effects on women with PCOS is of paramount importance due to the syndrome’s intricate hormonal imbalances and associated reproductive challenges. Furthermore, women experiencing HA, characterized by disrupted GnRH pulsatility, can benefit from triptorelin’s potential to restore endocrine balance. By elucidating the nuances of triptorelin’s effects on these distinct groups, researchers and clinicians can tailor its use for optimal therapeutic outcomes [8,9].

## Review

Endocrine profiles in healthy women

Normal Menstrual Cycle and Hormone Levels

The average menstrual cycle orchestrates an intricately timed sequence of hormonal fluctuations, spanning an average of 28 days, that serves as the cornerstone of female reproductive physiology. This rhythmic choreography is characterized by distinct phases governed by a coordinated interplay of hormones. The cycle commences with the follicular phase, an initial stage marked by the orchestrated orchestration of hormonal cues. At this juncture, the FSH takes center stage, propelling the growth and maturation of ovarian follicles. Within the ovaries, granulosa cells are spurred into action, synthesizing and secreting estradiol, a vital estrogen hormone. This surge in estradiol levels, akin to a crescendo, sets the stage for a profound surge in LH release [10,11].

The culmination of this hormonal symphony is ovulation, a climactic event where a matured ovarian follicle achieves its zenith and ultimately releases an egg, the potential harbinger of new life. The orchestrated surge of LH plays a pivotal role in this process, precisely timed to prompt the rupture of the follicle and release of the egg from the ovary. This carefully choreographed ballet of hormonal cues ensures that the ovulated egg is poised for potential fertilization, in line with the intricacies of the female reproductive cycle [12].

As the ovulatory curtain closes, the luteal phase takes the spotlight. During this interval, the remnants of the ovulated follicle metamorphose into the corpus luteum, an endocrine structure responsible for progesterone production. Progesterone, aptly called the “hormone of pregnancy,” is instrumental in orchestrating the uterine environment to implant a fertilized egg potentially. The uterine lining, known as the endometrium, is meticulously primed and thickened, creating a nurturing milieu that could foster the development of an embryo [13].

This intricately woven narrative of hormonal symphony underscores the awe-inspiring complexity of the normal menstrual cycle. Every hormonal cue, every surge and dip, is a meticulously timed note in this symphonic composition that orchestrates the potential for life. The follicular, ovulation, and luteal phases harmoniously collaborate to create the cyclical melody central to the rhythms of female fertility and reproductive health [14].

Role of the Hypothalamus-Pituitary-Gonadal Axis

The HPG axis is a cornerstone of the intricate hormonal symphony orchestrating reproductive processes. This complex axis, which comprises the hypothalamus, pituitary gland, and gonads, serves as a finely tuned regulatory system that governs the cyclic release of crucial sex hormones and holds paramount importance in maintaining reproductive function. The orchestration begins with the hypothalamus, a small yet vital brain region that operates as the conductor of this hormonal symphony [15].

The hypothalamus executes its role by precisely releasing GnRH, a potent signaling molecule that guides the pituitary gland’s actions. The release of GnRH is marked by its characteristic pulsatile pattern, emphasizing the intricacy and precision of this orchestration. This rhythmic release pattern of GnRH plays a pivotal role in maintaining the delicate balance of hormonal feedback loops [16].

Upon reaching the pituitary gland, GnRH acts as a catalyst, prompting the release of two key gonadotropins: LH and FSH. LH and FSH, collectively known as gonadotropins, venture to the ovaries, the gonadal powerhouse in females. These gonadotropins interface with their respective receptors within the ovaries, effectively stirring the production and release of sex hormones [17].

Estradiol and progesterone, the primary sex hormones in females, are the crescendos of this harmonious hormonal symphony. Estradiol, a potent estrogen, is pivotal in promoting the growth and maturation of ovarian follicles, which are sacs containing the developing eggs. It reaches its peak levels during the follicular phase of the menstrual cycle, playing a central role in stimulating the LH surge that triggers ovulation [18].

Impact of Triptorelin on Healthy Women’s Endocrine Profiles

Triptorelin’s synthetic analog nature enables precise manipulation of the HPG axis. Triptorelin’s administration initially causes a paradoxical surge in LH and FSH in healthy women, mimicking the natural GnRH stimulation. However, this effect is transient, as continued administration leads to receptor desensitization and downregulation of LH and FSH release. This suppression reduces estradiol and progesterone levels, leading to anovulation and menstrual cycle disruption [19].

Triptorelin’s controlled suppression of gonadotropin release finds application in assisted reproductive technologies, where controlled ovarian hyperstimulation is necessary for optimizing oocyte retrieval. Furthermore, it offers therapeutic potential in conditions requiring hormone-sensitive suppression, such as endometriosis or uterine fibroids. Understanding the nuances of triptorelin’s impact on healthy endocrine profiles contributes to its broader applicability in clinical practice [20].

Polycystic ovary syndrome and endocrine disruptions

Introduction to Polycystic Ovary Syndrome and Diagnostic Criteria

PCOS is a multifaceted endocrine disorder with profound implications for reproductive-age women. It is a condition marked by a complex interplay of hormonal imbalances and reproductive irregularities that transcend mere gynecological concerns. The intertwining triad of hyperandrogenism, ovulatory dysfunction, and polycystic ovarian morphology is central to PCOS’s intricate manifestation [21].

Hyperandrogenism, the elevation of male hormone levels such as testosterone, contributes to the clinical androgenic manifestations observed in PCOS. This can encompass hirsutism, characterized by excessive facial and body hair growth, acne, and alopecia. Ovulatory dysfunction, a core feature of PCOS, leads to irregular menstrual cycles or even anovulation, where ovulation fails to occur. This irregularity disrupts fertility and contributes to the complex clinical picture of PCOS [22].

One of the defining characteristics of PCOS, often visible through ultrasound imaging, is the presence of polycystic ovaries. These ovaries are characterized by multiple small follicles that appear like a “string of pearls” on ultrasound. However, it is important to note that not all individuals with PCOS will exhibit classic ovarian morphology, and the diagnostic criteria extend beyond this hallmark feature [23].

To standardize the diagnosis of PCOS, the Rotterdam criteria have gained widespread acceptance. These criteria stipulate that for a diagnosis of PCOS to be established, at least two of the following must be present: irregular menstrual cycles, clinical or biochemical signs of hyperandrogenism, and polycystic ovaries on ultrasound. This approach acknowledges the heterogeneous nature of PCOS presentations and highlights the importance of assessing multiple aspects of the syndrome to reach a comprehensive diagnosis [24].

Altered Hormone Levels and Menstrual Irregularities in Polycystic Ovary Syndrome

PCOS is a complex endocrine disorder that intricately impacts hormone levels and disrupts the delicate balance within the reproductive system. One of the hallmark features of PCOS is the presence of elevated androgen levels, particularly testosterone. This androgen excess contributes to physical symptoms such as hirsutism (excessive hair growth) and acne, affecting individuals’ appearance and emotional well-being [21].

Central to the hormonal dysregulation in PCOS is the HPG axis dysfunction. This axis orchestrates the menstrual cycle by coordinating the release of crucial hormones that stimulate the ovaries and regulate ovulation. In PCOS, the intricate dance of this axis becomes disrupted, leading to anovulatory cycles in which ovulation does not occur. The ovaries’ sensitivity to gonadotropins such as LH and FSH is reduced, further compounding the irregularities in hormone levels and ovulation [25].

Another pivotal aspect of PCOS is the frequently coexisting insulin resistance, wherein the responsiveness of the body’s cells to insulin actions diminishes. This physiological state gives rise to hyperinsulinemia, characterized by elevated insulin levels in the bloodstream. This, in turn, plays a significant role in the augmentation of ovarian androgen production, specifically testosterone synthesis. The intricate interaction between insulin resistance and the surplus of androgens establishes a self-perpetuating cycle. The heightened levels of insulin act as a stimulant for the ovaries to enhance the generation of androgens, thereby sustaining the hormonal imbalances that define PCOS [26].

Effects of Triptorelin on Endocrine Markers in Polycystic Ovary Syndrome

Triptorelin’s potential impact on individuals with PCOS holds particular intrigue due to its unique ability to modulate the intricate web of the HPG axis and the associated hormone secretions. Research has shed light on the intricate mechanisms through which triptorelin administration could alleviate the multifaceted hormonal disruptions characteristic of PCOS [27].

Triptorelin’s administration in PCOS patients has shown promising results in normalizing LH and FSH levels. This normalization represents a pivotal step in restoring the delicate equilibrium between these two gonadotropins, which are often dysregulated in PCOS. Restoring LH and FSH balance has significant implications for ovarian function, potentially fostering a more favorable environment for ovulation. By fine-tuning the pulsatile release of these hormones, triptorelin may act as a catalyst in reestablishing the natural ovulatory processes often compromised in PCOS [28].

Moreover, triptorelin’s effects extend to androgen regulation, the cornerstone of PCOS pathology. By suppressing the release of LH and FSH, triptorelin indirectly dampens the ovarian production of androgens, such as testosterone. This reduction in androgen levels can potentially mitigate the hyperandrogenic symptoms that frequently plague individuals with PCOS, including hirsutism and acne. The interconnected nature of the endocrine system means that triptorelin’s impact on gonadotropin suppression can indirectly address the androgen excess that is a hallmark of PCOS [29].

Potential Therapeutic Implications of Triptorelin in Polycystic Ovary Syndrome Management

The intricate hormonal imbalances characteristic of PCOS have spurred interest in triptorelin as a potential therapeutic intervention. Triptorelin’s controlled manipulation of endocrine profiles offers a promising avenue for addressing the multifaceted challenges posed by PCOS [30].

PCOS, marked by hyperandrogenism, anovulation, and insulin resistance, underpins various reproductive and metabolic dysfunctions. Triptorelin’s mechanism of action, involving transient gonadotropin release followed by downregulation, holds the potential to restore ovulatory cycles, a central concern for PCOS patients desiring conception. By orchestrating a regulated pattern of gonadotropin release, triptorelin may help normalize follicular development and ovulation, enhancing the chances of a successful pregnancy [31].

Moreover, triptorelin’s impact on androgen levels presents an intriguing dimension for PCOS management. Elevated androgens contribute to hirsutism, acne, and alopecia, significantly affecting the quality of life. Triptorelin’s suppression of gonadotropins indirectly leads to reduced ovarian androgen production, potentially alleviating these cosmetic concerns. Additionally, as insulin resistance exacerbates PCOS symptoms, triptorelin’s role in regulating hormonal balance might contribute to metabolic improvements [32].

However, while the potential therapeutic implications of triptorelin in PCOS management are promising, several aspects require further elucidation. Establishing optimal dosing regimens and treatment durations specific to PCOS is paramount to balancing achieving desirable outcomes and minimizing potential side effects. Furthermore, a comprehensive investigation into the long-term effects of triptorelin in PCOS is essential, ensuring that its benefits are sustained over time and potential risks are thoroughly understood [33].

Hypothalamic amenorrhea and endocrine dysregulation

Hypothalamic Amenorrhea: Causes and Consequences

HA is a perplexing disorder characterized by the conspicuous absence of menstrual periods due to intricate disruptions within the delicate HPG axis. Often targeting women with reduced energy availability, strenuous exercise regimes, or elevated stress levels, HA unveils the remarkable sensitivity of the female reproductive system to physiological stressors. The origins of HA are frequently woven from a complex tapestry of interrelated factors, with a convergence of inadequate caloric intake, intense physical exertion, and psychosocial stressors driving the disorder’s manifestation [34].

The repercussions of HA extend far beyond the realm of fertility, echoing through multiple facets of a woman’s physiological well-being. The absence of menstruation reflects the underlying disarray within the HPG axis, where diminished GnRH pulsatility engenders hormonal dysregulation. Amidst this turmoil, the impaired secretion of LH and FSH disrupts the orchestrated dance of ovarian follicle development, eventually leading to anovulation [35].

Yet, HA’s impact reaches beyond reproductive concerns, leaving an indelible imprint on broader health domains. The compromised estrogen production stemming from the anovulatory state strikes at the heart of bone health, rendering women vulnerable to decreased bone mineral density and osteoporosis, a condition fraught with long-term implications for skeletal integrity. Furthermore, the muted estrogen milieu in HA reverberates through cardiovascular corridors, potentially heightening the risk of adverse cardiac events. The repercussions even stretch into the landscape of psychological well-being, potentially fostering mood disturbances and altered cognitive function [36].

In the tapestry of women’s health, HA emerges as a complex thread, intertwining intricate hormonal disruptions with sweeping consequences for diverse physiological systems. The recognition of HA’s multifaceted nature underscores the imperative for a holistic approach that restores reproductive function and attends to the broader canvas of well-being [37].

Disrupted Hypothalamus-Pituitary-Gonadal Axis and Hormone Imbalances in Hypothalamic Amenorrhea

HA is characterized by an intricate interplay of endocrine disruptions that reverberate across the HPG axis, exerting far-reaching effects on hormonal balance and reproductive function. Within this context, the HPG axis, which orchestrates the pulsatile release of GnRH and its downstream effects, becomes dysregulated in HA, giving rise to intricate hormone imbalances [38].

Central to this disruption is the diminished GnRH pulsatility, a hallmark of HA. The hypothalamus, responsible for generating these pulsatile signals, experiences a blunting of its GnRH release pattern, leading to reduced frequency and amplitude of GnRH pulses. This dampened GnRH output translates into diminished LH and FSH secretion from the pituitary gland. This hormonal derangement results in a cascade of downstream effects reverberating through the entire reproductive system [39].

In the context of ovarian function, the suppressed LH and FSH levels hinder the growth and maturation of ovarian follicles. The reduced stimulation from these hormones results in insufficient development of follicles, thwarting the ovulatory process. This anovulatory state deprives women of the potential to release mature eggs, contributing to infertility [40].

Moreover, the decreased ovarian activity translates into inadequate estrogen production, a hormone pivotal for many physiological functions beyond reproduction. The implications of estrogen deficiency extend to bone health, where reduced estrogen levels can precipitate a decline in bone mineral density. Compromised bone strength elevates the risk of long-term osteoporosis and fractures [41].

The suppression of reproductive hormones in HA reverberates beyond reproductive and bone health. It impacts various physiological systems, including the cardiovascular, metabolic, and psychological. The absence of estrogen’s protective effects on cardiovascular health may contribute to increased cardiovascular risk factors. Metabolic perturbations such as altered glucose metabolism and lipid profiles may also arise. The hormonal imbalance could also manifest as psychological disturbances, including mood fluctuations and cognitive changes [42].

Exploring the Role of Triptorelin in Restoring Endocrine Balance in Hypothalamic Amenorrhea

The mechanism underlying HA is intricate, involving the disruption of the HPG axis and subsequently leading to the absence of menstrual cycles. This multifaceted condition frequently emerges due to factors such as energy deficiency, excessive exercise, or psychosocial stress. These triggers set off a complex series of endocrine imbalances that have extensive ramifications. In light of this, the investigation into triptorelin’s potential role in addressing HA has gained significant momentum. This is attributed to triptorelin’s unique and meticulously orchestrated mechanisms [35], which offer a compelling avenue for exploration.

Triptorelin’s initial paradoxical effect on gonadotropin release, followed by downregulation, is particularly intriguing when applied to HA. The transient simulation of LH and FSH release, akin to natural GnRH pulsatility, holds the promise of restoring the disrupted hormonal rhythms observed in HA. By initiating these pulses, triptorelin may trigger a cascade of events, including reinstating proper GnRH signaling. This, in turn, could promote more regular menstrual cycles, a crucial aspect of reproductive health beyond fertility concerns [43].

One of the paramount implications of triptorelin’s potential role in HA is the enhancement of estrogen production. The suppressed gonadal activity in HA results in diminished estrogen levels, posing adverse effects on bone health, cardiovascular function, and overall well-being. If successful, triptorelin’s modulation of the HPG axis could lead to the restoration of estrogen production. This, in turn, may alleviate the adverse consequences of estrogen deficiency, particularly the heightened risk of osteoporosis and other associated health issues [44].

The ramifications of triptorelin’s application in HA extend beyond reproductive health, encapsulating broader well-being. While the primary focus may be restoring ovulation and menstrual regularity, the potential impact on hormonal balance reverberates through various physiological systems. If successful, triptorelin could emerge as a tool for fertility enhancement and holistic health improvement in HA individuals [35].

Considerations for Triptorelin Use in Hypothalamic Amenorrhea Treatment

Triptorelin presents a promising avenue for HA treatment, potentially restoring the disrupted GnRH pulsatility and consequently reestablishing menstrual regularity. However, a thoughtful assessment of various considerations is imperative before embracing triptorelin as a therapeutic solution [8].

Addressing the underlying causes of HA is paramount. HA often arises from factors such as energy deficiency, excessive exercise, and psychosocial stress. These factors are pivotal in suppressing GnRH pulsatility and disrupting the HPG axis. Therefore, while triptorelin can potentially restore the endocrine balance, a comprehensive approach encompassing lifestyle modifications, nutritional counseling, and stress reduction strategies should be integrated alongside pharmacological interventions. Treating the root causes with triptorelin ensures a holistic and sustainable outcome [45].

The optimal dosing and duration of triptorelin treatment necessitate careful calibration. Balancing the objective of reinstating menstrual regularity with the potential risks is critical. Overstimulation or extended suppression of the HPG axis could lead to unintended consequences or inadequate outcomes. Tailoring triptorelin dosing regimens to individual needs, considering factors such as age, the severity of HA, and responsiveness to treatment, is essential to strike an optimal balance between restoration of fertility and minimizing potential adverse effects [46].

In treating HA with triptorelin, vigilance regarding potential side effects is paramount. As with any pharmaceutical intervention, there is a spectrum of potential reactions. Monitoring bone health is of particular concern, as HA’s disruption of the hormonal milieu may lead to decreased bone density. Triptorelin’s impact on estrogen levels and its subsequent influence on bone health warrants close observation. Additionally, side effects such as hot flashes and mood changes accompanying hormonal therapies should be monitored and managed effectively to ensure a positive treatment experience [47].

Comparative analysis of triptorelin effects

Comparing Endocrine Responses in Healthy, Polycystic Ovary Syndrome, and Hypothalamic Amenorrhea Women

Triptorelin’s multifaceted impact on endocrine profiles is magnified when considering its effects across distinct populations: healthy women, those with PCOS, and individuals experiencing HA. These three groups represent a spectrum of endocrine regulation, allowing for a comprehensive assessment of triptorelin’s capabilities and limitations. By delving into these diverse responses, a more nuanced understanding of the drug’s effects emerges, illuminating its potential applications and highlighting its adaptability [48].

In healthy women, triptorelin’s introduction triggers a brief yet intense surge in LH and FSH. This surge, akin to the natural pulsatile pattern of GnRH release, is swiftly followed by receptor desensitization and subsequent downregulation of the HPG axis. This effect, observed in response to triptorelin, mirrors the fine-tuned hormonal fluctuations of a healthy menstrual cycle [17].

Unlike the typical endocrine profile, individuals with PCOS exhibit a distinctive hormonal landscape marked by heightened androgen levels and irregular menstrual patterns. This specific endocrinological aberration characterizes PCOS. The role of triptorelin in this context is notably profound. While its primary mechanism entails the inhibition of LH and FSH release, it also engenders the normalization of these pivotal hormones. This restorative normalization can potentially reestablish ovulatory function and mitigate the hyperandrogenic symptoms observed in PCOS. This multifaceted triptorelin action directly addresses the disorder’s fundamental aspects [49], underscoring its significance in PCOS management.

Individuals grappling with HA present yet another complex endocrine scenario. HA is marked by disruptions in GnRH pulsatility, suppressing LH and FSH release, and ovulatory dysfunction. Triptorelin’s role in this population lies in its potential to restore proper GnRH pulsatility, a process crucial for reinstating regular menstrual cycles and fertility. By leveraging its ability to transiently stimulate LH and FSH release, triptorelin may facilitate the re-establishment of healthy hormonal rhythms, offering hope for HA individuals seeking to regain their reproductive health [50].

Identifying Commonalities and Disparities in Triptorelin’s Impact

Delving into the effects of triptorelin across diverse populations, healthy individuals, those with PCOS, and individuals experiencing HA, unveils a complex tapestry of commonalities and disparities. This comparative analysis is a powerful lens to peer into the intricate mechanisms of triptorelin’s actions and extrapolate its clinical implications [8].

Commonalities resonate at the core of triptorelin’s influence. The universal thread woven through these populations is the suppression of gonadotropin release, primarily LH and FSH. This suppression cascades downward, leading to decreased production of sex hormones, such as estrogen and progesterone. This shared effect underpins triptorelin’s utility as a modulator of endocrine balance, irrespective of the underlying health condition [51].

Disparities emerge as distinct brushstrokes across the canvas of triptorelin’s impact. While the fundamental outcome, reduction in sex hormone levels, may be consistent, the nuances of this impact vary. In the context of PCOS, triptorelin offers the prospect of restoring ovulatory function milestones unattainable for healthy individuals. Furthermore, restoring proper hypothalamic function and subsequent GnRH pulsatility in HA is a unique hallmark of triptorelin’s potential [52].

These disparities extend to the degree of suppression and the duration of effects. Healthy individuals may experience a transient yet significant gonadotropin surge, whereas the degree of suppression might be more pronounced in conditions characterized by excess hormone production, such as PCOS. Additionally, the duration of triptorelin’s effects could span from the duration of administration to more extended periods, presenting both therapeutic windows and challenges [22].

The implications of identifying these commonalities and disparities are far-reaching. Researchers and clinicians gain insights into these effects’ underlying mechanisms by deciphering the intricate web of triptorelin’s actions. Moreover, this understanding lays the groundwork for developing targeted interventions. Tailoring triptorelin’s use to the specific needs of each group promotes ovulation in PCOS, restoring GnRH pulsatility in HA, or achieving controlled hyperstimulation in assisted reproduction revolutionizes endocrine management [53].

Implications for Personalized Treatment Approaches

The intricate analysis of triptorelin’s effects across distinct populations enhances our understanding of its mechanisms and lays the foundation for tailoring treatment strategies to individual health conditions. This paradigm shift toward personalized approaches holds significant implications for optimizing therapeutic outcomes [54].

For healthy women seeking assisted reproduction, the controlled ovarian hyperstimulation induced by triptorelin becomes a strategic tool. By synchronizing follicular development and enhancing the chances of multiple viable oocytes, triptorelin offers a targeted approach to maximize the success of assisted reproductive techniques. This individualized intervention aligns with this group’s needs and goals, contributing to more effective fertility treatments [55].

In PCOS, a condition characterized by complex hormonal imbalances, triptorelin’s potential to restore hormonal equilibrium and promote ovulation emerges as a beacon of hope. Tailoring treatment for PCOS patients using triptorelin addresses the root causes of the disorder, thereby improving menstrual regularity and reducing the detrimental effects of hyperandrogenism. This individualized approach in PCOS management enhances reproductive prospects and alleviates the associated metabolic and cosmetic concerns [56].

Mechanisms of triptorelin action on endocrine profiles

Mechanism of Gonadotropin-Releasing Hormone Agonists

GnRH agonists, with triptorelin as a prominent example, constitute a class of pharmacological agents meticulously designed to intricately modulate the HPG axis. These synthetic analogs are strategically developed to leverage structural similarities with the native GnRH, the pivotal regulator of reproductive hormonal pathways. Notably, while these synthetic counterparts retain structural parallels, they exhibit divergent pharmacokinetic profiles, which underlie their distinctive impacts on the endocrine system [57]. The nuanced interplay between their molecular architecture and pharmacokinetics critically shapes their physiological effects.

The frequency and administration route of pulsatile stimulation in GnRH agonists are crucial to address. This mechanism involves a precise interplay between controlled suppression and pulsatile stimulation. These agonists are administered in a pulsatile manner by mimicking the natural pulsatile secretion pattern of GnRH in the hypothalamus. This carefully orchestrated approach initiates the activation of GnRH receptors, leading to a surge in the release of LH and FSH from the pituitary gland. This, in turn, replicates the physiological gonadotropin surge necessary for facilitating ovulation within a healthy menstrual cycle [17].

However, the intrigue deepens when considering prolonged exposure to GnRH agonists in dosage and duration. Upon continuous administration, an intriguing paradox emerges, receptor desensitization. The sustained stimulation of GnRH receptors leads to their desensitization, reducing the pituitary gland’s responsiveness to GnRH agonists. This finely orchestrated process results in a gradual decrease in LH and FSH release over time. Given the pivotal roles of LH and FSH in driving gonadal activity, their dampened effects due to GnRH agonists ultimately suppress gonadal function [58].

This controlled suppression and desensitization mechanism underpins the therapeutic potential of GnRH agonists such as triptorelin. Their capacity to temporarily mimic the physiological surge in gonadotropins, followed by the subsequent downregulation, allows for precise manipulation of endocrine profiles. This versatility has profound implications, from fertility enhancement in assisted reproduction techniques to ameliorating hormone-driven conditions such as hormone-sensitive cancers. Thus, the mechanism of GnRH agonists unveils a delicate balance between stimulation and suppression, presenting a strategic tool for modulating the intricate web of hormonal regulation within the HPG axis [59].

Triptorelin’s Impact on Gonadotropin Release and Gonadal Function

Triptorelin’s influence on gonadotropin release and gonadal function is a pivotal aspect of its mechanism of action. This influence is mediated through its intricate interaction with GnRH receptors in the hypothalamus and pituitary gland. Upon initial administration, triptorelin initiates a complex cascade of events that temporarily mirrors the natural pulses of GnRH secretion. This paradoxical effect rapidly increases LH and FSH, mimicking the characteristic preovulatory hormonal surge [60].

However, this surge is not sustained. With the continuation of triptorelin administration, a shift occurs in the responsiveness of pituitary receptors. The prolonged exposure to triptorelin ultimately triggers the desensitization of these receptors. As a result, the pituitary gland enters a refractory state, rendering it less sensitive to GnRH signals. This, in turn, leads to a gradual reduction in LH and FSH secretion, ultimately dampening the ovarian response and suppressing gonadal activity [61].

The controlled downregulation of the HPG axis detailed in this section bears profound therapeutic implications. The precision with which hormonal release can be modulated holds substantial promise across various clinical scenarios. Notably, with its ability to effectively lower gonadotropin levels, triptorelin assumes a pivotal role in treating hormone-dependent cancers. By diminishing the supply of sex hormones that drive the proliferation of these malignancies, triptorelin exerts a therapeutic effect that has been increasingly recognized. Furthermore, the controlled ovarian hyperstimulation facilitated by triptorelin’s transient surge of gonadotropins has a significant role in assisted reproduction. This process lays the groundwork for optimizing procedures such as oocyte retrieval and in vitro fertilization, as highlighted in prior research [62]. Adding to its spectrum of applications, triptorelin emerges as a multifaceted agent, demonstrating its potential to manage an array of malignancies while contributing to advanced reproductive techniques.

Modulation of Hormonal Signaling Pathways by Triptorelin

Triptorelin’s impact on endocrine profiles extends beyond its effects on the HPG axis, encompassing a range of downstream hormonal signaling pathways. Notably, this peptide exerts its influence by meticulously modulating specific pathways, yielding diverse physiological outcomes. In the context of hormone-related disorders such as PCOS, triptorelin’s suppression of gonadotropin release and subsequent reduction in sex hormone levels play a pivotal role. This orchestrated intervention mitigates the repercussions of excessive hormone production commonly observed in such conditions. Moreover, triptorelin’s intricate adjustments to estrogen and progesterone levels carry systemic implications, reverberating through domains such as bone health, cardiovascular function, and metabolic equilibrium [8].

Beyond its implications for reproductive health, triptorelin’s remarkable capacity to manipulate hormonal signaling pathways holds promise for broader therapeutic applications. An illustrative example lies in its potential to curtail the progression of hormone-sensitive cancers. By limiting the availability of hormones through suppressing gonadotropins, triptorelin emerges as a strategic contender in impeding cancer growth. Furthermore, triptorelin’s nuanced regulation of estrogen levels can yield therapeutic advantages in managing conditions intricately linked to hormone balance [62]. This multifaceted mechanism of action underlines triptorelin’s significance as a versatile agent with far-reaching physiological impacts.

Future directions and implications

Unanswered Questions and Research Gaps

Despite significant progress in understanding triptorelin’s effects on endocrine profiles, several questions and research gaps still need to be answered. One key area is the long-term impact of triptorelin use on bone health and cardiovascular function, particularly in populations prone to these concerns, such as women with HA. Additionally, optimal dosing regimens and treatment durations for specific conditions such as PCOS require further investigation. The interplay of triptorelin with other therapeutic interventions, such as lifestyle modifications and hormonal supplementation, also warrants exploration.

Potential Applications Beyond Polycystic Ovary Syndrome and Hypothalamic Amenorrhea

While triptorelin’s effects on PCOS and HA have garnered attention, its potential extends to other conditions involving endocrine dysregulation. For instance, in endometriosis, where hormonal imbalances contribute to symptomatology, triptorelin’s capacity to suppress gonadotropins could offer relief. Similarly, its role in managing hormone-sensitive cancers, such as breast or prostate cancer, is an area ripe for exploration. These potential applications highlight the versatility of triptorelin across a spectrum of endocrine-related conditions.

Integrating Triptorelin Into Comprehensive Endocrine Management

As research unfolds, integrating triptorelin into comprehensive endocrine management strategies holds promise. Tailoring its use to individual patient profiles, including age, hormonal levels, and underlying conditions, is essential for optimizing outcomes. Combining triptorelin with other modalities, such as nutritional counseling, exercise regimens, or hormonal therapies, could yield synergistic effects, particularly in complex conditions such as PCOS and HA. Collaborative efforts between endocrinologists, reproductive specialists, oncologists, and other relevant disciplines will be instrumental in developing holistic treatment approaches.

## Conclusions

In conclusion, triptorelin emerges as a dynamic tool that can shape the intricate landscape of endocrine regulation. Its precise mechanism of transient stimulation and subsequent downregulation of the HPG axis provides a nuanced approach to manipulating hormonal balance. Triptorelin’s effects unfold uniquely across diverse populations, ranging from healthy individuals to those grappling with conditions such as PCOS and HA. This underscores the significance of tailoring treatment strategies to the specific health condition, accounting for the variations in endocrine responses. Numerous questions and research gaps beckon as we traverse uncharted territories, inviting exploration into triptorelin’s long-term implications, optimal dosing regimens, and potential applications beyond its current scope. The promise of triptorelin, however, is undeniable. It can potentially revolutionize women’s health by restoring ovulatory function, mitigating hormonal imbalances, and fostering a comprehensive approach to endocrine management. With every investigation, we move closer to unlocking triptorelin’s full potential and its role in reshaping the landscape of hormonal equilibrium, thereby elevating the realms of reproductive health and well-being for women.
